# A True Thrombosed External Jugular Vein Aneurysm Presenting as a Cervical Mass: A Case Report and Literature Review

**DOI:** 10.7759/cureus.25757

**Published:** 2022-06-08

**Authors:** Kaisor Iqbal

**Affiliations:** 1 Division of Vascular Surgery, Department of Surgery, College of Medicine, King Saud University Medical City, King Saud University, Riyadh, SAU

**Keywords:** venous thrombosis, ultrasound, valsalva maneuver, external jugular vein, aneurysm

## Abstract

External jugular venous aneurysms are a rare clinical occurrence. Clinical symptoms such as painless neck swelling that worsens with the Valsalva maneuver, coughing, or straining may point to the diagnosis. Doppler ultrasonography is frequently used to confirm the diagnosis.

We report a case of a 43-year-old woman who presented with a one-year history of painless right supraclavicular swelling. She noticed the swelling increases in size with the Valsalva maneuver. Neck ultrasound with color Doppler revealed a vascular structure containing turbulent blood flow; it measured 2.9 x 1.2 cm and appeared partially thrombosed. During further evaluation at our hospital, the patient underwent a computed tomography scan of the neck with intravenous contrast, which revealed a right supraclavicular mass measuring 2.3 x 3.5 cm and likely representing an external jugular vein saccular aneurysm. The patient underwent surgical excision. Histopathological examination of the external jugular vein aneurysm revealed vascular tissue with blood clots, hemosiderin-laden macrophages, and attached benign fibroadipose tissue. The postoperative recovery was uneventful, and the patient was discharged home with regular follow-up in the last one year in our outpatient clinic.

Venous aneurysms are an uncommon clinical phenomenon. Saccular venous aneurysms, which mostly affect adults and involve the external jugular venous and internal jugular vein, have degenerative histology and are more prone to thrombosis. Asymptomatic and uncomplicated jugular venous aneurysms should be treated conservatively with regular follow-up. Enlarging, disfiguring, symptomatic, and complicated jugular venous aneurysms, on the other hand, almost always necessitate surgical exclusion and bypass.

In comparison to arterial aneurysms, true venous aneurysms are relatively uncommon. For saccular jugular venous aneurysms, surgical excision is the gold standard and is indicated to reduce the risk of aneurysmal rupture and pulmonary embolism.

## Introduction

Overall, venous aneurysms are a rare clinical phenomenon [1-3], and Harris was the first to characterize them in 1928 [4]. They have a benign clinical presentation and only cause pain if they are found in the head and neck area. They may appear with embolism or rupture instead of those found in other locations [2]. Clinical presentation such as a compressible, soft, painless neck swelling that increases with the Valsalva maneuver or straining may indicate the presence of a jugular venous aneurysm. Imaging modalities such as ultrasound with color Doppler, computed tomography scan, and magnetic resonance imaging are frequently used to confirm the diagnosis [5-7]. We report a case of a 43-year-old woman with a true external jugular vein aneurysm presented as a lateral neck mass, which was diagnosed using ultrasound Doppler, confirmed by computed tomography scan, and was surgically resected.

## Case presentation

**Figure 1:**
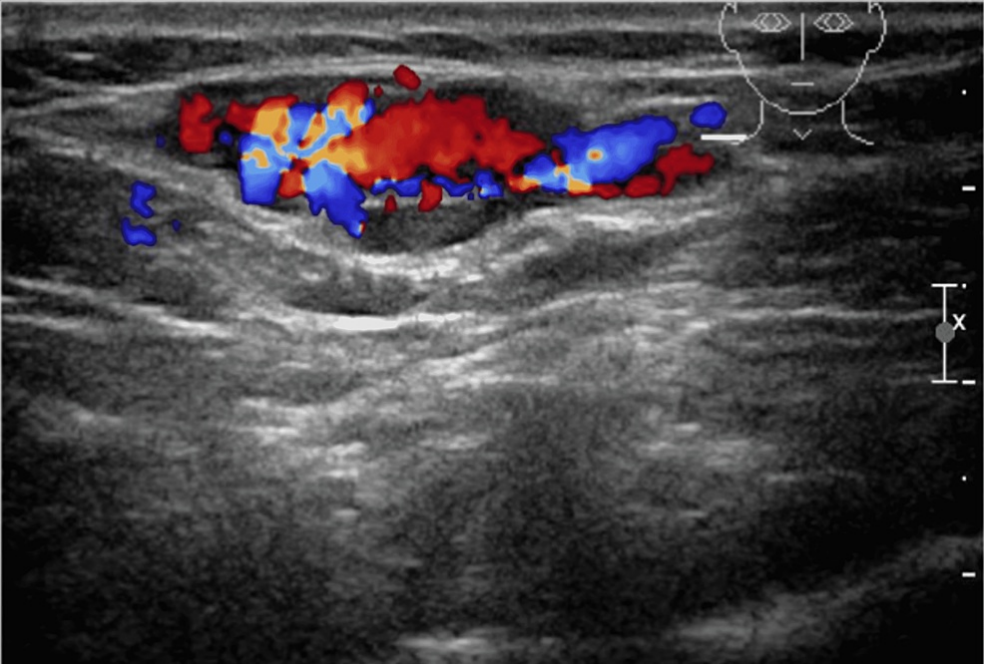
Ultrasound of the neck with color Doppler revealed a vascular structure containing turbulent blood flow. It measured 2.9 x 1.2 cm and appeared partially thrombosed with communication with the venous system.

**Figure 2:**
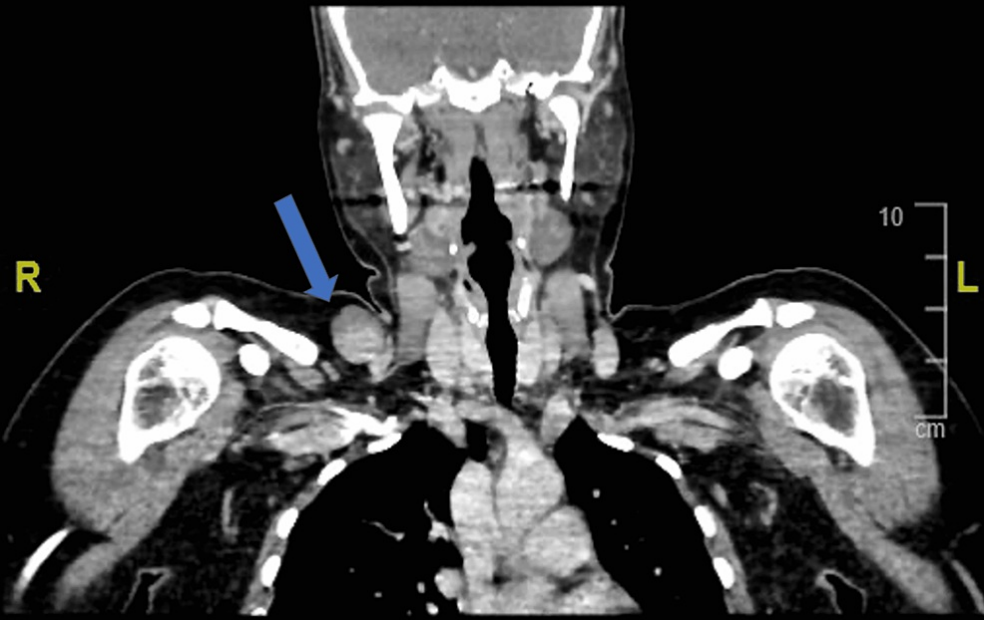
Computed tomography scan of the neck with intravenous contrast (coronal view) revealed a right supraclavicular mass measuring 2.3 x 3.5 cm (arrow). It showed close relation to the right external jugular vein with short neck communication, and it likely represented an external jugular vein saccular aneurysm.

**Figure 3:**
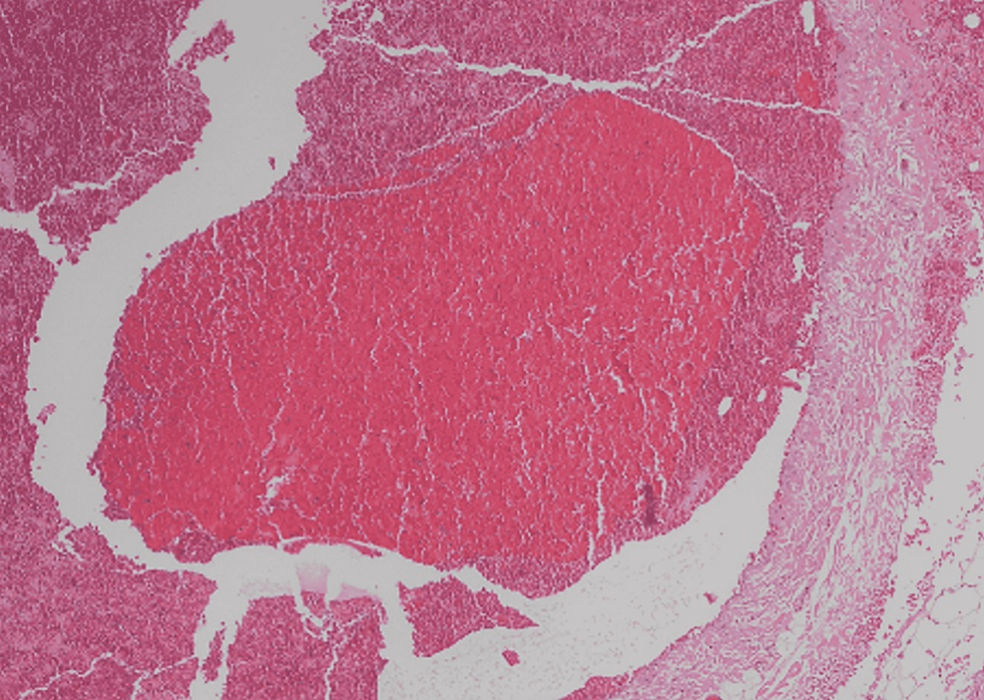
Histologic section of the aneurysm showed an organized blood clot in the lumen and a thinned muscular wall.

A 43-year-old woman with no history of any medical illness was referred to our vascular surgery outpatient department with complaints of painless right supraclavicular swelling for one year. The swelling was insidious in onset and gradually increased over time. She noticed the swelling increases in size with the Valsalva maneuver. She denied any history of pain, fatigue, anorexia, diarrhea, jaundice, and weight loss. Also, the patient denied any history of trauma to the neck region. The past surgical history and family history were unremarkable. Upon general physical examination, the patient was not in pain and had no pallor or jaundice. She was vitally stable and afebrile. Local examination revealed a soft, non-tender, compressible, and non-pulsatile swelling measuring approximately 3 x 2 cm on the right side of the neck at the supraclavicular region. The swelling was well prominent by the Valsalva maneuver. No bruit was audible on auscultation. Laboratory investigations showed neither leukocytosis nor neutrophilia. Other laboratory results were unremarkable. Neck ultrasound with color Doppler revealed a vascular structure containing turbulent blood flow; it measured 2.9 x 1.2 cm and appeared partially thrombosed. Communication with the venous system was noted (Figure 1). On further evaluation of the right supraclavicular lesion, a computed tomography scan of the neck with intravenous contrast revealed a right supraclavicular lesion measuring 2.3 x 3.5 cm. It showed close relation to the right external jugular vein with short neck communication. The lesion was non-enhancing in the arterial phase with progressed incomplete enhancement in the venous phase. In correlation with the previous ultrasound neck images, it likely represented an external jugular vein saccular aneurysm (Figure 2). The decision was made to take the patient to the operation room. Intraoperatively, there was a 2 x 3 cm saccular aneurysm arising from the external jugular vein, and resection of the aneurysm was performed. The histologic section of the aneurysm showed an organized blood clot in the lumen and a thinned muscular wall (Figure 3). The postoperative recovery was uneventful. The patient was discharged home in good condition and remained asymptomatic during the regular follow-up in the last one year post-operation in our outpatient clinic.

## Discussion

In comparison to arterial aneurysms, true venous aneurysms are relatively uncommon [8]. Lower extremity venous aneurysms account for about 77% of all venous aneurysms, followed by the internal jugular vein (approximately 13%) and 10% in the upper extremities [9]. Saccular venous aneurysms of the external jugular vein are extremely rare, with only a few cases reported in the English literature, and they can be classified as true venous aneurysms [10]. Saccular venous aneurysms have degenerative histology and a higher risk of thrombosis, as seen in our case, and are most commonly found in adults involving the external jugular vein and internal jugular vein [9,11]. A saccular aneurysm of the jugular vein usually manifests itself clinically as a painless swelling [12]. Arteriovenous malformation, retention cysts, cystic hygroma, cupula inflation, laryngocele, carcinomas of surrounding organs, enlarged lymph node, and other cancers tumors are among the differential diagnosis for lateral neck mass [9]. A venous aneurysm is defined by the presence of a non-tender, soft, unilateral, and non-pulsatile swelling that enlarges with straining or the Valsalva maneuver [12]. During the physical assessment of our case, these signs were discovered. The radiological studies used to establish the diagnosis range from simple ultrasound to advanced diagnostic tools like computed tomography scan and magnetic resonance imaging [5]. For asymptomatic and uncomplicated jugular venous aneurysms, conservative management with frequent follow-up is suggested. Surgical exclusion and bypass are frequently required for enlarging, disfiguring, symptomatic, and complicated jugular venous [13]. For saccular jugular venous aneurysms, surgical resection is the gold standard [9,14]. Surgical resection decreases the risk of aneurysmal rupture and pulmonary embolism. Furthermore, a case of a thrombosed external jugular vein aneurysm causing unsuspected pulmonary embolisms was recently reported, indicating that those aneurysms may not be as harmless as initially believed [15]. In our case, surgical excision was performed.

## Conclusions

External jugular vein saccular venous aneurysms are relatively uncommon. A venous aneurysm is characterized by a non-tender, soft, unilateral, and non-pulsatile swelling that enlarges with straining or the Valsalva maneuver. Imaging modalities such as the ultrasound with color Doppler or a computed tomography scan are frequently used to confirm the diagnosis. Among the potential diagnoses of lateral painless neck swelling that worsens on Valsalva maneuver or straining, external jugular vein aneurysms require a high suspicion index. The gold standard for reducing the risk of aneurysmal rupture and pulmonary embolism is surgical excision.
